# The association between subjective health perception and lifestyle factors in Shiga prefecture, Japan: a cross-sectional study

**DOI:** 10.1186/s12889-020-09911-y

**Published:** 2020-11-25

**Authors:** Sae Tanaka, Sayu Muraki, Yuri Inoue, Katsuyuki Miura, Eri Imai

**Affiliations:** 1 Department of Nutrition, School of Human Cultures, The University of Shiga Prefecture, Hikone City, Japan; 2 Department of Public Health Care and Welfare, Shiga Prefectual Headquarters, Otsu, Shiga Japan; 3 Department of Public Health, Shiga University of Medical Science, Otsu, Shiga Japan

**Keywords:** Subjective health, Diet, Lifestyle behavior, Longevity

## Abstract

**Background:**

The Ministry of Health has reported that in Japan, the Shiga prefecture has the highest life expectancy. Subjective health perception is a predictive indicator of mortality. For this study, we examined the association between subjective health perception and multiple lifestyle factors.

**Methods:**

Data were obtained from the 2015 Health and Nutrition Survey in Shiga prefecture. The analytic sample comprised 6057 adults aged 20 or older. Information on subjective health perception and lifestyle behaviors was obtained from a self-administered questionnaire. As for subjective health perception, participants were divided into 2 groups: (1) Excellent or Good and (2) Average, Poor, or Very Poor. A 1-day dietary survey was also administered. The health behaviors score (HBS) was calculated based on 5 factors: consuming a healthy diet, never smoking, low-risk alcohol drinking, regular exercise, and moderate sleep duration. HBS scores ranged from 0 to 5. Multiple logistic regression was used to calculate the sex-, age- BMI- and energy intake-adjusted odds ratios (ORs) of poor subjective health across HBS, with 0 points as the reference.

**Results:**

Among all participants, 2397 (39.6%) individuals were classified into the good subjective health group. Participants with an HBS of 3 (OR 0.59, 95% CI 0.37–0.96), 4 (OR 0.40, 95% CI 0.24–0.65) or 5 (OR 0.33, 95% CI 0.19–0.59) had a lower OR of rating themselves as being average/poor health compared with those having zero. The association with a higher HBS was remarkable (*p* for trend: < 0.001). Additional analyses revealed that the combinations including regular exercise were particularly associated with a lower risk of subjective average/poor health.

**Conclusions:**

This study showed that the higher the number of healthy lifestyle factors, the lower risk of subjective average/poor health. Combinations of healthy lifestyle factors, especially those involving exercise, suggest good subjective health for individuals living in the Shiga prefecture.

## Background

Life expectancy in Japan is higher than in other developed countries [1]. The Ministry of Health has reported that life expectancy in Japan is 81.09 years for men, which is second only to Switzerland, and for women, it is the longest in the world at 87.26 years. According to the Ministry of Health, Shiga prefecture has the highest life expectancy in Japan for men, at 81.8 years, and the fourth highest for women, at 87.6 years, which slightly lower than Nagano, Okayama and Shimane prefecture [2]. The trend of life expectancy in Shiga from 1965 to 2015 revealed yearly increases for both sexes [3].

There are reports that in Japan, increased life expectancy is due to decreased mortality from cardiovascular diseases and neoplasms or cancers [4]. In fact, the standardized mortality ratio of cerebrovascular diseases and cancers in Shiga were lower than in other Japanese prefectures [5].

Studies have revealed that lifestyle factors are independently associated with the risks of all-cause and cause-specific mortality. A meta-analysis has reported that current smoking is a risk factor of all-cause and lung cancer death [6] On the other hand, and a high intake of fruits and vegetables reduces the risks of all-cause, cardiovascular diseases, and cancer mortality [7]. These lifestyle behaviors tend to be concurrent in individuals [8]. Moreover, accumulating evidence has shown that the greater the number of healthy lifestyle factors, the lower the risk of mortality, and the lower the number of healthy lifestyle factors, the higher the risk of mortality [9–15]. A large cohort study investigating the association between the risk of mortality and certain lifestyle factors has reported that the combination of a greater number of factors is associated with increased survival rate [12].

Subjective health perception often used in an overall measure of health status, consists of one simple question. This indicator has been found predictive of all-cause and cause-specific mortality. A large body of evidence has shown that those with a poor subjective health perception had a higher risk of mortality than those with a good subjective health perception [16–20]. As for longevity, in the prospective study followed for 30 years, the survival curve showed those with an excellent subjective health perception lived longer than those with poor/very poor subjective health perception [21]. These evidences have suggested that the life expectancy of the individuals perceiving their subjective health as good will be long. A study considering subjective health perception as an outcome would be helpful in identifying those at a high risk of early-stage mortality.

Some study have revealed that subjective health perception is associated with lifestyle factors [22–27]. An R et al. have reported that participants who consumed vegetables and fruits almost every day and meat occasionally were more likely perceive their subjective health to be good than those who did not [23]. Longitudinal research has revealed current smokers or previous smokers, especially men, were more likely to perceive themselves to be in poor health [22]. However, to our knowledge, only 4 studies have explored the effect of combined lifestyle factors in terms of subjective health perception [28–31]. The 3 studies of them evaluated dietary consumption from a limited number of food groups [28–30]. Because people consume foods in combination, studying the overall diet is useful for promoting dietary recommendations to the public. In addition, recent prospective studies have suggested that higher diet quality reduced the risk of mortality [32]. Additionally, the participants in two of the studies were invited from among those who had registered for a physical activity promotion initiative [29, 30], limiting the generalizability of the research results. Another study assessed only 3 behaviors and Body Mass Index (BMI) [31]. A more general and comprehensive study is therefore needed, assessing multiple lifestyle behaviors including an overall diet quality. Furthermore, the question of which combinations of behaviors are particularly associated with subjective health perception has not yet been revealed. This study aimed to examine how the combination of five lifestyle factors—consuming a healthy diet, never smoking, low-risk alcohol drinking, regular exercise, and moderate sleep duration—were associated with subjective health perception in general adults in Shiga prefecture, Japan.

## Methods

### Data source

The Health and Nutrition Survey of Shiga prefecture (HNSS) is a survey that covers approximately 4000 households, once every 5 years, in randomly selected census units defined by the Shiga prefecture. It provides the largest prefecturally representative sample available by which to monitor dietary intake and lifestyle factors of the people in the Shiga prefecture. The present study was approved by the Shiga prefecture in Japan. The most recent data in 2015, covering 4229 households, were analyzed in the study. The HNSS was conducted on one day in November 2015.

### Participants

**Fig. 1:**
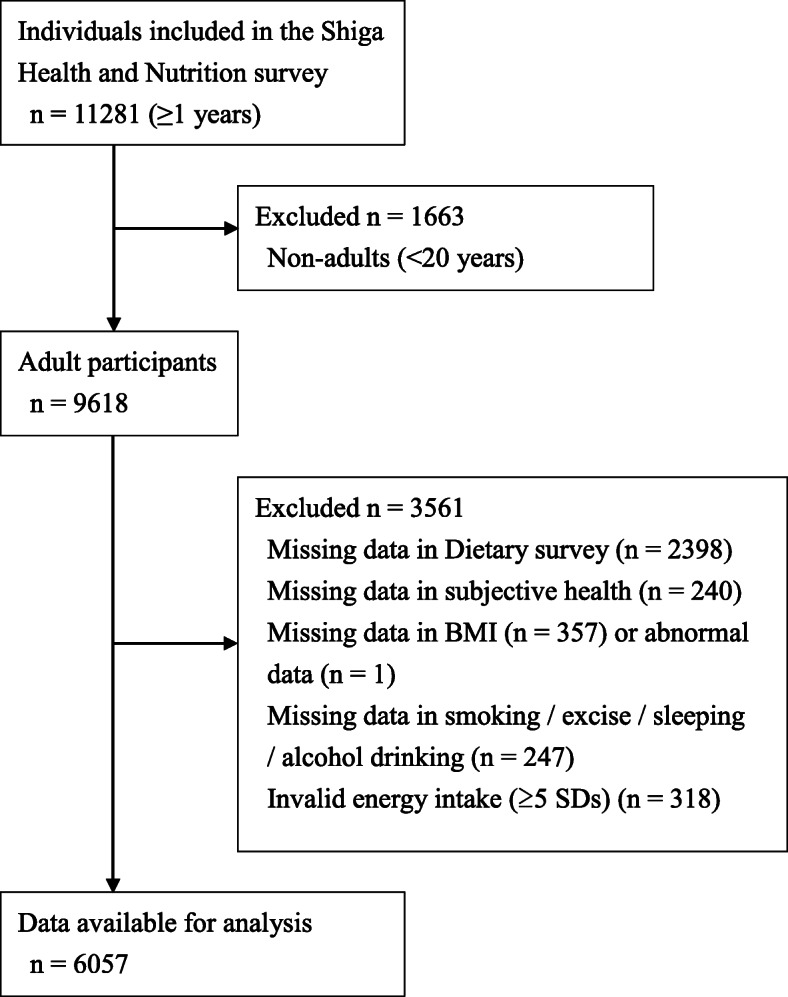
A flow diagram of individuals included in the study

Data were the HNSS obtained from, Japan, in 2015. Figure 1 shows a flow diagram of individuals included in the study. The survey included 4229 households chosen randomly from approximately 1% of the households of all 19 cities in Shiga, and involved all household members aged ≥1 year. A total of 1663 individuals aged < 20 years were excluded. In addition, a total of 2398, 240, 358, and 247 individuals who provided incomplete data for the survey on diet, health status, BMI, and lifestyle factors (smoking, exercise, sleeping, and alcohol drinking), respectively, were also excluded. Then, 318 individuals with an implausibly high- or low-energy intake (in the upper or lower 2.5% of energy intake distribution, respectively) were excluded from the study. Ultimately, data from 6057 individuals (2790 men, and 3267 women, mean age ± standard deviation (SD) 55.0 ± 16.9 years) were analyzed. The 3561 individuals aged ≥20 years, whose data excluded from the study sample, did not differ from the study sample in terms of distribution of sex (Men = 47.2%), BMI (22.6 ± 3.6 kg/m^2^) and energy intakes (1817 ± 896 kcal/d). Age were significantly higher (55.9 ± 18.5 years) and subjective health perception were poor (“excellent” and “good” = 37.2%); however, the difference between the groups was not large.

### Ethics statement

The present analysis was based on a secondary analysis of observational survey data. No ethical review was sought based on the Ethical Guidelines for Medical and Health Research Involving Human Subjects, given this study used only information that had already been anonymized. The HNSS, conducted by the government of Shiga prefecture, has stringent protocols and procedures that ensure confidentiality and protect individual participants from being identified. The Shiga prefecture government anonymized all individual-level data before providing the authors with the datasets for this study.

### Measurements

#### Subjective health perception

We used a single item to measure general health: “How is your current health? (1) Excellent, (2) Good, (3) Average, (4) Poor, (5) Very Poor.” Those who answered excellent or good were classified into the good subjective health group, and those who answered average, poor or very poor were classified into the average or poor subjective health group [22].

#### Diet quality

The nutrient and food intake were estimated by a 1-day household dietary record. The record included the approximate proportions the dish was divided into among the family members to estimate the energy, nutrient, and food intakes of each individual. The energy and nutrient intakes of each individual were calculated using the Japanese Standard Food Composition Table [33]. Energy and macronutrient intakes estimated using this method highly correlated with the intake estimated using individualized diet records (Pearson’s correlation coefficients: r = 0.90 for energy, 0.89 for protein, 0.91 for total fat, and 0.90 for carbohydrate) [34]. The quality of the diet was assessed with the reference values given in the Dietary Reference Intakes for Japanese 2015 (DRIs-J) [35] and modified the previously developed score [36, 37]. The 18 nutrients assessed in the study included protein, total fat, carbohydrate, vitamin A, vitamin B_1_, vitamin B_2_, niacin, vitamin B_6_, vitamin B_12_, folic acid, vitamin C, calcium, magnesium, iron, zinc, copper, dietary fiber, and saturated fatty acid. For each of the 18 nutrients, a score of 1 was allocated if the nutrient met or exceeded the recommended daily allowance (RDA) or tentative dietary goal (DG) given in DRIs-J and a score of 0 if it did not meet RDA or DG. DG was defined as the current goals for Japanese individuals to reach the average daily intake of nutrients for the prevention of lifestyle-related diseases in the DRIs-J 2015. It was determined based on the results of many nutritional epidemiology studies. Additionally, the nutrients having a tolerable upper intake level (UL) were given a score of 1 if the nutrient was below UL, and 0 if it was above the UL. For the 18 nutrients included, we used the RDA for 14 nutrients (protein, vitamin A, vitamin B_1_, vitamin B_2_, niacin, vitamin B_6_, vitamin B_12_, folic acid, vitamin C, calcium, magnesium, iron, zinc and copper) and the DG for 4 nutrients (total fat, carbohydrate, dietary fiber, and saturated fatty acid). Energy intake was assessed using the Estimated Energy Requirement (EER). EER = basal metabolic reference value (kcal/kg body weight/day) × body weight (kg) × physical activity level (PAL). Basal metabolic reference values and PAL were given in DRIs-J 2015. Body weight of each individual were used. A score of 1 was allocated if an individual’s energy intake was ≥ EER with PAL = 1 and ≤ EER with PAL = 3, and 0 if it deviated from the range. The range of PAL values were 1.50–2.00 for participants aged 20–69 years and 1.45–1.95 for participants aged ≥70 years. In DRIs-J 2015, PALs of 1, 2, and 3 were considered low, moderate, and high, respectively, and can be characterized as a sedentary lifestyle, sedentary work including housework and light-intensity sports, and work with high-intensity physical activity or leisure-time physical activity, respectively. The total DRIs-J score was constructed by summing the scores of the 18 nutrients and energy intake. The DRIs-J scores ranged from 0 to 19. Each participant was given 1 point for a DRIs-J score greater than or equal to the median score by sex in the participants (the median score was 7.0 for both sexes).

#### Lifestyle factors

Information on height, body weight, smoking, exercise, sleeping, alcohol drinking was obtained from the self-administered questionnaire. BMI was calculated as body weight (kg) divided by height squared (m^2^). Smoking was assessed with three options: current, former, or never smoker. Participants who never smoked were given 1 point. Current and former smokers were scored 0 to avoid misclassification for those who stopped smoking recently. Information on alcohol drinking was obtained regarding the frequency and quantity by using 2 questions. The frequency of alcohol drinking was reported with seven options: “never”, “in the past”, “occasionally”, “1–2 times per week”, “3–4 times per week”, “5–6 times per week”, and “every day”. If participants answered “never”, “in the past”, or “occasionally” for the first question, they were scored 1. If participants consumed alcoholic beverages 1–2 times per week or more, a second question was asked about the average quantity consumed per day. Alcohol consumption ≥40 g/d for men or ≥ 20 g/d for women was scored 0. Alcohol consumption < 40 g/d for men or < 20 g/d for women was scored 1. Excessive amounts of alcohol have been reported to be associated with a higher risk of lifestyle-related diseases [38–40], and alcohol consumption < 40 g/d for men and < 20 g/d for women has been officially recommended in Japan [41].

Exercise was assessed with 2 questions. First, individuals were asked “How often do you regularly do physical activity consciously for your health?” Participants chose from 4 options: “never”, “in the past”, “sometimes”, and “usually”. If participants answered “never” or “in the past” for the first question, they were scored 0.

Those who answered “sometimes” and “usually” then answered the following question: “Have you done 30 minutes of exercise at least twice a week for a year?” The participants chose from three options: “never”, “in the past”, and “current”. Those who answered “sometimes” or “usually” for the first question and “current” for the second question were given 1 point, whereas those who answered “sometimes” or “usually” for the first question and “never” or “in the past” for the second question were scored 0. The amount of exercise equal to 4 metabolic equivalents (METs) hour per week has been reported to be associated with a low risk of all-cause and cancer mortality [13, 42] and is recommended in the Japanese national health promotion: Health Japan 21 (the second term) [41].

Sleep duration was assessed using the question, “How many hours do you usually sleep per day?” The response categories were: < 5 h/d, ≥ 5 h/d and < 6 h/d, ≥ 6 h/d and < 7 h/d, ≥ 7 h/d and < 8 h/d, ≥ 8 h/d and < 9 h/d, ≥ 9 h/d. Those who answered ≥7 h/d and < 8 h/d or ≥ 8 h/d and < 9 h/d were given 1 point. The rest were scored 0. This duration has been suggested to have an association with a low risk of all-cause mortality [43].

#### Health behaviors score

We established health behaviors score (HBS) referring to previous studies [10–12]. Table 1 shows the scoring of health behaviors in the study. The HBS was estimated based on whether individuals met each of 5 criteria: diet quality, smoking, alcohol drinking, exercise behavior, and sleeping. The HBS ranged from 0 to 5, with a higher score indicating a higher number of good lifestyle behaviors.

**Table 1:** Scoring of health behaviors in the study

Health behaviors	Scoring method
Diet quality	1 = DRIs-J score ^a^ ≥ median specified by sex in the Individuals. (7.0 for both sexes)
Smoking	1 = never smoked.
Alcohol drinking	1 = consuming alcohol < 40 g/d for men or < 20 g/d for women.
Exercise behavior	1 = engaging in 30 min of exercise at least twice a week and continuing for a year.
Sleeping	1 = sleeping for ≥7 h/d and < 9 h/d.
Total score	5

^a^ Dietary Reference Intakes for Japanese score (ranging from 0 to 19)

### Statistical analysis

To compare the characteristics of the individuals according to their subjective health perception, t tests and a χ^2^ test were used for mean values and SD for continuous variables, and percentages for categorical variables. Multiple logistic regression was used to investigate the association between subjective health perception (two groups, good and average or poor health perception) and the HBS. The odds ratios (ORs) and 95% confidence intervals (CIs) for average/poor subjective health perception were calculated with the HBS as the exposure variable. Next, to examine the specific patterns of health behaviors, variables representing possible combinations of the HBS were created. Some variables were not included in the analysis because they were rarely observed in this population. These models were adjusted for sex, age (continuous) and BMI (continuous) based on previous studies that examined the relationship between subjective health perception, death and lifestyle factors [12, 14, 15, 25], and energy intake (continuous). Energy intake differed between individuals with a good subjective health perception and those with an average/poor subjective health perception (*p* <  0.001); the higher the dietary score, the higher the energy intake (Pearson’s correlation coefficient: r = 0.632). We tested for interactions by introducing a multiplicative term into the main effect models; however, there was no change in the statistical significance of the results. For all analyses, statistical significance was defined as a two-tailed *p*-value of < 0.05. All statistical analyses were performed using SAS software (version 9.4; SAS Institute, Cary, NC, USA).

## Results

A total of 6057 individuals were included in the analysis. Of them, 39.6% reported their subjective health perception as “Excellent” or “Good”. Table 2 shows the characteristics of the individuals according to their subjective health perceptions. Compared with participants in the average or poor subjective health group, those in the good subjective health group were younger, had a lower BMI, a better diet based on DRIs-J, more moderate sleep duration, and were more likely to exercise regularly, although the deference of BMI was small. When assessing sleep duration, this study population was more likely to have insufficient sleep rather than excess sleep (73.3% for the good subjective health group and 75.5% for the average or poor subjective health group).

**Table 2:** The characteristics of the individuals by subjective health perception

	Average or poor subjective health group ^a^	Good subjective health group ^b^	*p* value
	*n* = 3660	*n* = 2397	
Men (%)	46.6	45.2	0.266
Age (years)	55.7 ± 16.7 (58.0)	53.9 ± 17.1 (57.0)	< 0.001
BMI (kg/m^2^)	22.6 ± 3.4 (22.3)	22.3 ± 2.9 (22.1)	< 0.001
Energy intakes (kcal/d)	1759 ± 465 (1718)	1808 ± 472 (1766)	< 0.001
Health behaviors score (points) ^c^	2.6 ± 1.0 (3.0)	2.8 ± 1.0 (3.0)	< 0.001
Health behaviors (%)			
DRIs-J score ≥ median in the study ^d^	56.7	60.2	0.006
Never smoking	62.4	62.7	0.806
Consuming alcohol < 40 g/d for men or < 20 g/d for women	93.1	92.4	0.336
Engaging in 30 min of exercise at least twice a week and continuing for a year	21.0	37.2	< 0.001
Sleeping for ≥7 and < 9 h/d	22.4	25.2	0.012

Values are mean ± SD, (median), or percent

*p* value < 0.05 was significant

^a^ The individuals with “Average”, “Poor” or “Very poor”

^b^ The individuals with “Excellent” or “Good”

^c^ Health behaviors score was consisted of 5 health behaviors in this table

^d^ The value was 7.0 for both sexes

Table 3 shows the association between multiple lifestyle factors and subjective health perception. In unadjusted models, compared with those with an HBS of 0, those with an HBS of 1, 2 and 3 did not have significantly lower odds of rating themselves as being in average/poor health. However, those with an HBS of 4, or 5 had a lower OR of average/poor subjective health (OR 0.44, 95%CI 0.27–0.72, *p* for trend < 0.001; OR: 0.38, 95% CI 0.22–0.67, *p* for trend < 0.001 respectively). After adjusting for sex, age, BMI and energy intake, compared with those with an HBS of 0, those with an HBS of 1 and 2 did not have significantly lower odds of rating themselves as being in average/poor health. However, those with an HBS of 3, 4, or 5 had a lower OR of rating themselves as being average/poor health (OR 0.59, 95% CI 0.37–0.96, *p* for trend = 0.033; OR: 0.40, 95% CI 0.24–0.65, *p* for trend < 0.001; OR 0.33, 95% CI 0.19–0.59, *p* for trend < 0.001, respectively). This association became stronger as the HBS increased (*p* for trend < 0.001).

**Table 3:** The association between health behaviors score and an average/poor subjective health perception

Health behaviors score^a^	n	Crude OR (95%CI)	Adjusted OR (95%CI) ^b^
0	85	1	1
1	704	0.82 (0.50–1.34)	0.82 (0.50–1.35)
2	1899	0.76 (0.47–1.22)	0.75 (0.46–1.21)
3	2142	0.62 (0.39–1.00)	0.59 (0.37–0.96) ^*^
4	1054	0.44 (0.27–0.72) ^***^	0.40 (0.24–0.65) ^***^
5	173	0.38 (0.22–0.67) ^***^	0.33 (0.19–0.59) ^***^

*OR* Odds ratio, *CI* Confidence interval

^a^ Health behaviors score was consisted of following 5 health behaviors, ranging from 0 to 5. Diet quality: DRIs-J score ≥ median by sex in the participants (7.0 for both sexes), smoking: never smoker, alcohol drinking: consuming alcohol < 40 g/d for men or < 20 g/d for women, exercise behavior: engaging in 30 min of exercise at least twice a week and continuing for a year and sleep: sleeping for ≥7 h/d and < 9 h/d

^b^ Adjusted for sex, age, BMI, and energy intake

^*^ *p* for trend < 0.05, ^***^ *p* for trend < 0.001

To examine which combinations of the HBS were associated with a lower risk of average/poor subjective health perception, we conducted further analyses of those with an HBS of 3 or higher. Table 4 shows the relationship between the combination of 3, or 4 HBS and subjective health perception. Of all 15 expected combinations, 5 combinations were rarely found; thus, 10 combinations were included. After adjusting for sex, age, BMI and energy intake, compared with 0 HBS, 5 combinations were significantly associated with a lower risk of having average/poor subjective health condition. All of the combinations included exercise behavior. Among those with an HBS of 3, exercise, better diet, and low-risk alcohol drinking showed the lowest OR of rating themselves as being average/poor health (OR 0.26, 95% CI 0.13–0.51, *p* for trend < 0.001). Among those with an HBS of 4, two combinations included exercise: exercise, better diet, low-risk alcohol drinking and never smoking and exercise, low-risk alcohol drinking, never smoking and moderate sleep duration were associated with a lower risk of average/poor subjective health (OR 0.34, 95%CI 0.18–0.64, *p* for trend < 0.001, OR 0.37, 95%CI 0.17–0.83, *p* for trend = 0.015 respectively). However, the association between exercise, better diet, low-risk alcohol drinking and moderate sleep duration and a risk of subjective average/poor health was not significant (OR 0.48, 95%CI 0.19–1.20, *p* for trend = 0.114). The results of investigating for individual health behaviors were shown in Additional file 1. Out of the five healthy behaviors, a person with an exercise score of 1 has the lowest probability of being evaluated as having an average/poor health condition than those with an exercise score of 0 (adjusted OR 0.40, 95% CI 0.35–0.45 *p* <  0.001).

**Table 4:** The association between the combinations of 3 or higher health behaviors and subjective health perception

Health behavior ^a^					n	Adjusted OR (95%CI) ^b^
Exercise	Better Diet	Low-risk Alcohol Drinking	Never Smoking	Moderate Sleep Duration		
0	0	0	0	0	85	Reference
1	1	1	0	0	275	0.26 (0.13–0.51) ^***^
1	0	1	1	0	211	0.35 (0.19–0.66) ^**^
1	0	1	0	1	54	0.40 (0.18–0.90) ^*^
0	1	1	0	1	171	0.87 (0.42–1.81)
0	1	1	1	0	1114	1.02 (0.59–1.77)
0	0	1	1	1	272	1.05 (0.57–1.96)
1	1	1	1	0	549	0.34 (0.18–0.64) ^***^
1	0	1	1	1	69	0.37 (0.17–0.83) ^*^
1	1	1	0	1	106	0.48 (0.19–1.20)
0	1	1	1	1	323	0.77 (0.39–1.52)

A “1” denotes the presence of the health behavior, and a “0” denotes the absence of the health behavior. OR: Odds ratio, CI: Confidence interval

^a^ Health behavior: Exercise; engaging in 30 min of exercise at least twice a week and continuing for a year, Better Diet; DRIs-J score ≥ median by sex in the participants (7.0 for both sexes), Low-risk Alcohol Drinking; consuming alcohol < 40 g/d for men or < 20 g/d for women, never smoking, and Moderate Sleep Duration; sleeping for ≥7 h/d and < 9 h/d

^b^ Adjusted for sex, age, BMI, and energy intake

^*^ *p* for trend < 0.05, ^**^ *p* for trend < 0.01, ^***^ *p* for trend < 0.001

## Discussion

The present study of adults revealed that the higher the number of healthy lifestyle factors, which comprised regular exercise, dietary adherence to DRIs-J, no excessive alcohol drinking, moderate sleep duration, and never smoking, the lower the risk of an average/poor subjective health perception. The combinations including exercise behavior were particularly likely to decrease the OR of subjective average/poor health perception.

We found that maintaining 3 or more healthy lifestyle behaviors was associated with lower risk of subjective average/poor health perception. Previous studies have shown that individual lifestyle-related behaviors are associated with subjective health perception. A longitudinal study reported that adults who smoked 10 or more cigarettes per day had a higher risk of rating themselves as having poor health compared with those who had never smoked after adjusting for potential confounders, including the participants’ disease conditions [44]. A large population survey revealed that those with higher physical activity or shorter sleep duration had a lower or higher adjusted risk ratio of rating themselves as having poor health, respectively [25]. These findings suggest that lifestyle-related factors are independently associated with subjective health perception and assist in interpreting the results of the present study. Subjective health perception has been shown to be a useful predictor of mortality [16–20]. Our finding has newly provided the notion that increasing the number of people engaging in combined lifestyle-related health behaviors should be an important goal of public health.

In the HBS used in our study, the combinations including exercise were strongly associated with a lower risk of subjective average/poor health perceptions. Furthermore, those with an exercise score of 1 had lower odds of rating themselves as having average/poor health compared with those with an exercise score of 0, which was the strongest association among the five health behaviors. In an overview examining epidemiological evidence for an association between physical activity and mental health, the potential anti-inflammatory effects of exercise, involving serotonin and noradrenaline, might be relevant at the neurobiological level as antidepressants and anxiolytics [45]. Double-blind randomized experiments in healthy men and women have reported that inflammatory activation is associated with declining subjective health perception [46]. Even considering the effect of exercise, the present study revealed that an HBS of 5 and combined behaviors, including exercise, had a greater reduction in the risk of poor subjective health than exercise alone. This supports our main finding that as more health behaviors are combined, the risk of poor subjective health perception is reduced.

On the other hand, in our study, the association between exercise, better diet, low-risk alcohol drinking and moderate sleep duration and a risk of subjective average/poor health was not significant. Of those engaged these 4 behaviors, 77.4% aged 65 or older, although the other individuals with an HBS of 4 included exercise did not have differences between the rate of 65 or older and under 65 years. Older people were more likely to perceive their subjective health poor than younger people [27]. In terms of the combination, never smoking was not included in the combination. Two prospective studies conducted over approximately 10-year periods revealed that among multiple lifestyle behaviors, physical activity displayed the strongest association with reduced mortality risk, followed by smoking [12, 14]. Although the present study did not identify an association between smoking behavior and subjective health perception, it has been suggested that people, particularly those in the older population, who smoked rated themselves as having poor health and that smoking weakened the effect of regular exercise.

There are some limitations to the study. First, because it was a cross-sectional study, we could not show a causal relationship between subjective health perception and the HBS. Second, the dietary record was conducted for only 1 day; thus, the dietary data might not reflect habitual intake. However, the nutrient intake of the individuals in our study was similar to Japanese habitual nutrient intake [47]. Third, measurements were self-reported and included somewhat vague scales, possibly leading to over- or under-reporting and misclassification. No information on intensity was available for the measurements of exercise intensity. However, a study of 483 Japanese adults reported that only answering a simple question about exercise habits, such as assessing step counts and the amounts of physical activity at 3 Mets or more, was sufficient for estimating physical activity levels [48]. Fourth, because of a loss of over 30% of the initial sample due to missing values in the key measures in this study, the individuals included in the analysis were not completely representative of the general population; for example, they may have high health awareness. Finally, we did not adjust for potential confounders such as income, educational level, disease conditions and mental health conditions because we could not obtain proper information from the prefecture-level health and nutrition survey. However, age, sex, BMI and energy intake, which we adjusted for in this study, are the most commonly adjusted factors among studies of the association between multiple lifestyle factors and mortality [12, 14, 15].

This study also has several strengths. The HBS consisted of the factors based on significant scientific evidence of an association with mortality risk. Subjective health perception is a useful measure for public health and nutrition assessment using a single survey question, and it has been validated as a tool to predict mortality in populations. To our knowledge, this is the first study of combinations of HBS and subjective health perception.

## Conclusions

The present study showed that 3 or more healthy lifestyle factors including regular exercise, high adherence to a healthy diet, moderate sleep duration, nonexcessive alcohol drinking, and never smoking were associated with a low risk of subjective average/poor health. The combinations of healthy lifestyle factors, particularly those that include exercise, indicate a good subjective health perception for individuals in the Shiga prefecture in Japan and widen the scope of choices available for people with poor subjective health.

## Supplementary Information

**Additional file 1. **The association between the individual health behaviors and an average/poor subjective health perception. OR: Odds ratio, CI: Confidence interval. ^a^ Health behavior: Better Diet; DRIs-J score ≥ median by sex in the participants (7.0 for both sexes), never smoking, Low-risk Alcohol Drinking; consuming alcohol < 40 g/d for men or < 20 g/d for women, Exercise; engaging in 30 min of exercise at least twice a week and continuing for a year, and Moderate Sleep Duration; sleeping for ≥7 h/d and < 9 h/d. ^b^ Adjusted for sex, age, BMI, energy intake and the other health behaviors included in this table. * *p* <  0.05, ^**^ *p* <  0.01, ^***^ *p* <  0.001.

## Data Availability

The government of Shiga prefecture anonymized individual-level data collected from the HNSS provided the corresponding author (Eri Imai) with the datasets for this study. Therefore, the datasets analyzed during the current study are not publicly available because of data protection, but the appropriate authors may be available dataset if reasonably request.

## Abbreviations

BMI: Body mass index
HNSS: Health and Nutrition Survey of Shiga prefecture
SD: Standard deviation
HBS: Health behaviors score
DRIs-J: Dietary reference intakes for Japanese
RDA: Recommended daily allowance
UL: Tolerable upper intake level
DG: Tentative dietary goal for preventing lifestyle-related diseases
EER: Estimated energy requirement
PAL: Physical activity level
METs: Metabolic equivalents
OR: Odds ratio
CI: Confidence interval
