# Supplementary material for: The association between subjective health perception and lifestyle factors in Shiga prefecture, Japan: a cross-sectional study
Source: BMC Public Health. 2020 Nov 25;20:1786. doi: 10.1186/s12889-020-09911-y (PMC7690120; doi:10.1186/s12889-020-09911-y)
Supplement: Supplementary file 1 — Additional file 1. The association between the individual health behaviors and an average/poor subjective health perception. OR: Odds ratio, CI: Confidence interval. a Health behavior: Better Diet; DRIs-J score ≥ median by sex in the participants (7.0 for both sexes), never smoking, Low-risk Alcohol Drinking; consuming alcohol < 40 g/d for men or < 20 g/d for women, Exercise; engaging in 30 min of exercise at least twice a week and continuing for a year, and Moderate Sleep Duration; sleeping for ≥7 h/d and < 9 h/d. b Adjusted for sex, age, BMI, energy intake and the other health behaviors included in this table. * p < 0.05, ** p < 0.01, *** p < 0.001. [file 12889_2020_9911_MOESM1_ESM.docx]

**Additional file 1. The association between the individual health behaviors and an average/poor subjective health perception.**

| Health behavior ^a^ |  | n | Crude OR  (95%CI) | Adjusted OR  (95%CI) ^b^ |
| --- | --- | --- | --- | --- |
| Better Diet | 0 | 2538 | 1 | 1 |
|  | 1 | 3519 | 0.86 (0.78–0.96) ^**^ | 0.98 (0.85–1.12) |
| Never Smoking | 0 | 2273 | 1 | 1 |
|  | 1 | 3784 | 0.98 (0.89–1.10) | 1.11 (0.97–1.28) |
| Low-risk Alcohol  Drinking | 0 | 436 | 1 | 1 |
|  | 1 | 5621 | 1.10 (0.90–1.34) | 1.14 (0.93–1.40) |
| Exercise | 0 | 4398 | 1 | 1 |
|  | 1 | 1659 | 0.45 (0.40–0.50) ^***^ | 0.40 (0.35–0.45) ^***^ |
| Moderate Sleep  Duration | 0 | 4631 | 1 | 1 |
|  | 1 | 1426 | 0.86 (0.76–0.97) ^*^ | 0.82 (0.73–0.93) ^**^ |

OR: Odds ratio, CI: Confidence interval

^a^ Health behavior: Better Diet; DRIs-J score ≥ median by sex in the participants (7.0 for both sexes), never smoking, Low-risk Alcohol Drinking; consuming alcohol < 40 g/d for men or < 20 g/d for women, Exercise; engaging in 30 min of exercise at least twice a week and continuing for a year, and Moderate Sleep Duration; sleeping for ≥ 7 h/d and <9 h/d.

^b^ Adjusted for sex, age, BMI, energy intake and the other health behaviors included in this table.

* *p* < 0.05, ^**^ *p* < 0.01, ^***^ *p* < 0.001
